# Incision Closure for Direct Anterior Total Hip Arthroplasty: Is There a Difference in the Rate of Superficial Wound Complications With Suture Versus Staples?

**DOI:** 10.7759/cureus.62145

**Published:** 2024-06-11

**Authors:** Arman C Hlas, Michael C Marinier, Ayobami S Ogunsola, Jacob M Elkins

**Affiliations:** 1 Department of Orthopedics and Rehabilitation, University of Iowa Hospitals and Clinics, Iowa City, USA

**Keywords:** surgical staples, skin suture, surgical wound complications, direct anterior approach, total hip arthroplasty

## Abstract

Background

Direct anterior total hip arthroplasty (DA-THA) has increased in popularity over recent decades. However, DA-THA has been reported to have a higher incidence of superficial wound complications, including infection and incisional dehiscence, compared to other surgical approaches to hip arthroplasty. While this indicates a need for optimal wound closure, little research exists on the preferred method of skin closure following DA-THA. This study aimed to determine if there was any difference in rates of superficial infection, wound dehiscence, or overall wound complications with skin closure using a running subcuticular 3-0 Monocryl® suture compared to surgical staples following DA-THA.

Methods

Records of patients who underwent DA-THA at our institution between July 2017 to July 2022 were retrospectively reviewed. Data were abstracted on patient demographics, comorbidities, skin closure method, and wound complications from the electronic medical record. Superficial infection and wound dehiscence were classified based on explicit diagnosis in post-operative records and incision photographs taken during follow-up visits. Overall wound complications were classified in patients who experienced either superficial infection, incisional dehiscence, or both complications following surgery. Descriptive statistics and chi-squared measures were obtained from post-operative patient data, and significance was set at p $\leq$ 0.05.

Results

A total of 365 DA-THAs were completed in 349 patients. A running subcuticular 3-0 Monocryl® suture closed 207 surgeries (56.7%), while surgical staples closed 158 surgeries (43.3%). There was no significant difference in independent rates of superficial infection (p = 0.076) or wound dehiscence (p = 0.118) between suture and staple cohorts; however, suture closure (10, 2.7%) was associated with a significantly higher rate of overall wound complications compared to staple closure (1, 0.3%) (p = 0.020).

Conclusion

DA-THA carries the risk of overall wound complications, including superficial infection and wound dehiscence. Our findings suggest superficial skin closure with staples may be preferred over sutures due to lower rates of overall wound complications. Further studies are needed to determine the optimal method of skin closure following DA-THA.

## Introduction

Direct anterior total hip arthroplasty (DA-THA) has grown in popularity over recent decades. The benefits of this approach include more rapid recovery times, decreased muscle damage and inflammation, and improved stability compared to other surgical approaches to hip arthroplasty [1-4]. However, DA-THA has been criticized for a high rate of superficial wound complications, including infection, incisional dehiscence, and other complications [5-12]. These can be a significant burden for both patients and orthopedic providers, as the rate of reoperation due to wound complications is seven times higher for DA-THA compared to the posterior approach [5].

DA-THA may predispose to wound complications due to incision proximity to the groin and its commensal microbiome, poor skin quality at the incision site, and impaired wound healing arising from hip movement separating the skin edges [6,13]. Surgical factors have also been found to play a role in the development of wound complications [14]. While this indicates a need for optimal wound closure following surgery, there is a paucity of information in the literature on the preferred method of skin closure and associated outcomes following DA-THA [5]. Across orthopedics, skin closure with sutures has generally been considered superior to staples due to decreased rates of post-operative wound complications and the improved aesthetic appearance of the resulting scar [15,16]. However, recent studies suggest there may be no difference in wound complications with staple closure compared to suture [17,18].

To our knowledge, there have been no studies evaluating the association between suture and staple skin closure on superficial wound complications following DA-THA. The goal of this study was to determine if there was any difference in rates of superficial infection, wound dehiscence, or overall wound complications with skin closure using a running subcuticular 3-0 Monocryl® suture compared to surgical staples following DA-THA. 

This article was presented as a poster at the American Academy of Orthopaedic Surgeons (AAOS) 2024 Annual Meeting on February 13, 2024, and at the 2024 Health Sciences Research Day at the University of Iowa Carver College of Medicine on April 26, 2024.

## Materials and methods

Study design

The study was conducted at the University of Iowa Hospitals and Clinics, Iowa City, United States. Using our institution’s electronic medical record, we accessed records from all patients who underwent DA-THA over five years from July 2017 to July 2022. Two orthopedic surgeons (JO and JE) who completed fellowship training in joint arthroplasty were included in this analysis, but only one of these surgeons (JE) is the author of this study. In total, 365 DA-THAs were completed in 349 patients over this period. 

Ethical considerations

This study was approved by the University of Iowa Institutional Review Board (IRB #201904825). 

Study criteria

A minimum of six-week patient follow-up was required as an inclusion criterion for the study. All 365 DA-THAs fulfilled this criterion and were therefore included in the analysis. Patients were to be excluded from the study if the method of superficial skin closure was not documented in the electronic medical record; however, this information was documented for each procedure, and no patients were excluded.

Surgical procedure

In all surgeries, a standard direct anterior approach was utilized [19]. All procedures concluded with identical joint irrigation using sterile 0.9% normal saline, capsular closure with a 1-0 Vicryl® suture, fascial closure with a running 2-0 Quill® suture, subcutaneous closure with a 0-0 Monocryl® suture, and deep dermal closure with a 2-0 Monocryl® suture. Superficial skin closure was completed with either a running subcuticular 3-0 Monocryl® suture or surgical staples, depending on the routine preference of the treating surgeon. Standard sterile wound dressings were applied following skin closure, and patients followed a standard post-operative protocol including perioperative IV antibiotics, deep vein thrombosis prophylaxis, and pain control as clinically indicated based on individual patient circumstances. 

Assessments

Pre-operative clinic notes were reviewed to gather patient baseline characteristics, including age, body mass index (BMI), gender, smoking status, and diabetes mellitus status at the time of surgery. Procedural notes were reviewed to identify the method of superficial skin closure. All post-operative records, including follow-up visits, telephone/telehealth visits, and other relevant patient communications in the electronic medical record, were reviewed to identify wound complications, including superficial infection and wound dehiscence, that occurred after surgery. Superficial infection and wound dehiscence were classified based on explicit diagnosis in post-operative records and by incision photographs taken during follow-up visits. Overall wound complications were classified in patients who experienced either superficial infection, wound dehiscence, or both complications following surgery.

Sample size calculations

The number of participants needed for this study was based on a priori power calculation using methods outlined by Serdar et al. [20]. Given the lack of literature on this topic, a conservative estimate of medium effect size was utilized. Using an estimated medium effect size (Cohen $\omega$ = 0.3), an alpha level of 0.05, and a power of 0.80, a sample size of 174 per group was needed to detect statistical significance. A failure to detect a difference across groups could represent a type II error.

Statistical analysis

Continuous variables were expressed as means $\pm$ standard deviations, and categorical variables were expressed as ratios based on cohort size. Differences in cohort baseline characteristics between patients closed with sutures versus staples were determined by t-tests and chi-squared measures. Descriptive statistics and chi-squared measures were additionally obtained from post-operative patient data. An alpha level of 0.05 was used to determine statistical significance. 

## Results

A total of 365 DA-THAs were included in this study. The study had 207 surgeries (56.7%) closed with a running subcuticular 3-0 Monocryl® suture, while 158 surgeries (43.3%) used surgical staples. The average patient age of those included in this study was 63.30 $\pm$ 12.06 years, and the average BMI was 27.88 $\pm$ 4.77 kg/m2. Table 1 outlines the baseline characteristics of patients in suture and staple cohorts. There were no significant differences in mean age, BMI, gender, smoking status, or diabetes mellitus status between these two cohorts.

**Table 1:** Patient baseline characteristics between the suture and staple cohorts.

	3-0 Monocryl_®_ (n = 207)	Staples (n = 158)	p-value
Age (years)	63.3	63.3	0.983
Gender (male/female)	112/95	73/85	0.135
Body mass index (kg/m^2^)	27.76	28.05	0.566
Diabetes mellitus	21	8	0.075
Tobacco use	16	7	0.199

Post-operative wound complications arose in 11 total surgeries (3.0%), including eight surgeries complicated by superficial infection (2.2%) and seven surgeries complicated by wound dehiscence (1.9%). There was no significant difference in the rate of superficial infection between patients closed with sutures (7, 1.9%) compared to staples (1, 0.3%) (p = 0.076). Additionally, there was no significant difference in the rate of wound dehiscence between patients closed with sutures (6, 1.6%) compared to staples (1, 0.3%) (p = 0.118). However, suture closure was associated with a significantly higher rate of overall wound complications (10, 2.7%) compared to staple closure (1, 0.3%) (p = 0.020) (Table 2).

**Table 2:** Wound complications and their associated method of skin closure.

	3-0 Monocryl_®_	Staples	p-value
Superficial infection	7 (1.9%)	1 (0.3%)	0.076
Incisional dehiscence	6 (1.6%)	1 (0.3%)	0.118
Overall wound complications	10 (2.7%)	1 (0.3%)	*0.020

## Discussion

The direct anterior approach offers numerous benefits compared to other approaches to total hip arthroplasty; however, DA-THA carries an elevated risk of superficial wound complications, including infection and incisional dehiscence. While these complications indicate a need for optimal wound closure following DA-THA, no previous studies have identified a preferred method of skin closure to reduce these complications. Our main objective was to determine if there was any difference in rates of superficial infection, wound dehiscence, or overall wound complications with skin closure using a running subcuticular 3-0 Monocryl® suture compared to surgical staples following DA-THA. Although we found no significant difference in independent rates of superficial infection or wound dehiscence between suture and staple cohorts, superficial skin closure with suture was associated with a significantly higher rate of overall wound complications compared to staple skin closure.

Wound complications after DA-THA

The risk of superficial wound complications, including infection and incisional dehiscence, remains a concern following DA-THA. Current estimates suggest the rate of these complications ranges from 1.2% to 4.6% of all surgeries [7,21]. In this study, the rate of superficial wound complications was 3.0%. These superficial complications can be challenging to manage post-operatively and can contribute to the development of further and more severe complications, including deep wound infection, periprosthetic joint infection, or implant failure [5,7,8,10]. Consequently, the rate of reoperation from wound complications is seven times higher for DA-THA compared to the posterior approach [5]. The direct anterior approach likely predisposes to wound complications due to intrinsic features of the surgery, including incisional proximity to the groin with its commensal microbiome, poor skin quality at the anterior hip near the incision site, and impaired wound healing arising from hip movement causing separation of the skin edges [6,13]. Major risk factors for these complications include obesity and diabetes mellitus, which is concerning as these conditions often present comorbidly in patients with osteoarthritis [12,22]. These distinct characteristics of DA-THA likely necessitate a greater need for optimal wound closure compared to other surgical approaches to hip arthroplasty.

Superficial skin closure with suture versus staples

Previous literature investigating rates of wound complications with suture and staple skin closure in orthopedics has been contradictory. Historically, suture skin closure has been considered superior to staple skin due to decreased rates of post-operative infection and the improved aesthetic appearance of the resulting scar [15,16,23,24]. However, more recent studies exploring suture versus staple skin closure have found no difference in rates of overall wound complications [17,18], and the method of skin closure should first optimize surgical outcomes and minimize complications over other considerations [16]. Several studies in the field of hip arthroplasty have investigated this further. Buttaro et al. found the complication rate of primary hip arthroplasty closed with staples to be higher than that of those closed with sutures, but this difference was not statistically significant [25]. Lu et al. reported that the complication rate of primary hip arthroplasty was significantly lower with continuous subcuticular sutures compared to both interrupted suture closure and staple closure [26]. Mallee et al. showed similar results, with the use of staples being associated with a nearly three-fold greater risk of surgical site infection compared to sutures [27]. As a result, international panels have recommended the use of subcuticular sutures over staples for total hip arthroplasty due to the lower risk of superficial infection and higher patient preferences [28]. However, these studies have not subdivided complications based on the specific approach used in hip arthroplasty; therefore, it is difficult to directly translate these findings to DA-THA. Given the unique features and risk of superficial wound complications with DA-THA compared to other approaches, this distinction is necessary to identify an optimal method of superficial wound closure. Our results demonstrate that skin closure with a suture carries a significantly higher rate of overall wound complications compared to staples. Surgical staples may offer benefits due to increased strength of wound closure, improved eversion of skin edges, and reduced tissue reaction and strangulation at the incision site [29]. To clarify and confirm these findings, further research is needed to investigate suture and staple skin closure and their associated outcomes following direct anterior total hip arthroplasty. 

Limitations

This study is not without limitations. First, the retrospective nature of this study relied on information documented in the electronic medical record, and a more intensive investigation of skin closure methods and superficial wound complications could not be completed. Second, our strict identification criteria for wound complications may have failed to capture other complications that can result from failed skin closure, including deep wound infection and periprosthetic joint infection, among others. This design was intended to ensure that identified complications were attributable to failure of superficial skin closure rather than failure of fascial, subcutaneous, or deep dermal closure. Third, our study was slightly underpowered by sixteen patients in the staple cohort, which may in part be due to our conservative estimate of medium effect size. Given the significant morbidity of these complications, in conjunction with the limited body of literature on this topic and the relatively small difference in sample size, we continue to believe these findings remain noteworthy for the field of arthroplasty. Fourth, our study only included two surgeons operating at a single institution, which introduces bias based on training and experience. To address this, we have outlined our specific approach to DA-THA, wound closure, and post-operative protocols so our findings can be interpreted in an appropriate context. Lastly, our study only examined complication rates among two different methods of superficial skin closure. Although superficial skin closure with a running subcuticular 3-0 Monocryl® suture or surgical staples is a routine preference at our institution, other closure methods exist but were not included in this study. 

## Conclusions

DA-THA carries the risk of superficial wound complications, including infection and incisional dehiscence. Our findings demonstrate that superficial skin closure with a running subcuticular 3-0 Monocryl® suture carries a significantly higher rate of overall wound complications compared to staple closure. Surgical staples may offer benefits due to increased strength of wound closure, improved eversion of skin edges, and reduced tissue reaction and strangulation at the incision site. Further studies are needed to investigate different methods of skin closure and identify an optimal method to reduce rates of superficial wound complications following DA-THA.
